# Microaneurysm Turnover in Mild Non-Proliferative Diabetic Retinopathy is Associated with Progression and Development of Vision-Threatening Complications: A 5-Year Longitudinal Study

**DOI:** 10.3390/jcm10102142

**Published:** 2021-05-15

**Authors:** Ana Rita Santos, Luis Mendes, Maria Helena Madeira, Ines P. Marques, Diana Tavares, João Figueira, Conceição Lobo, José Cunha-Vaz

**Affiliations:** 1 AIBILI—Association for Innovation and Biomedical Research on Light and Image, 3000-548 Coimbra, Portugal; asantos@aibili.pt (A.R.S.); lgmendes@aibili.pt (L.M.); mhmadeira@aibili.pt (M.H.M.); ipmarques@aibili.pt (I.P.M.); dstavares@aibili.pt (D.T.); joaofigueira@oftalmologia.co.pt (J.F.); clobo@aibili.pt (C.L.); 2 Department of Orthoptics, School of Health, Polytechnic of Porto, 4200-072 Porto, Portugal; 3 Faculty of Medicine, Coimbra Institute for Clinical and Biomedical Research (iCBR), University of Coimbra, 3000-548 Coimbra, Portugal; 4 Center for Innovative Biomedicine and Biotechnology (CIBB), University of Coimbra, 3000-548 Coimbra, Portugal; 5 Department of Ophthalmology, Centro Hospitalar e Universitário de Coimbra (CHUC), 3000-075 Coimbra, Portugal

**Keywords:** microaneurysm, diabetic retinopathy, severity progression, biomarker

## Abstract

Background: Analysis of retinal microaneurysm turnover (MAT) has been previously shown to contribute to the identification of eyes at risk of developing clinically significant complications associated with diabetic retinopathy (DR). We propose to further characterize MAT as a predictive biomarker of DR progression and development of vision-threatening complications. Methods: 212 individuals with type 2 diabetes (T2D; ETDRS grades 20 and 35) were evaluated annually in a 5-year prospective, longitudinal study, by color fundus photography and optical coherence tomography. Endpoints were diabetic macular edema (DME) or proliferative retinopathy (PDR). MAT analysis included determination of MA formation and disappearance rates, automatically assessed using the RetMarkerDR^®^. Retinopathy severity progression was evaluated using step increases in ETDRS severity levels. Results: Of the 212 individuals, 172 completed the 5-year follow-up study or developed an endpoint (n = 27). MAT calculated at 1 year showed a significant difference between groups of endpoint developments (*p* = 0.018), particularly MA disappearance rate (*p* = 0.007). MAT also showed a significant difference between eyes with different ETDRS severity progression in the 5-year period (*p* = 0.035). Conclusions: MAT is an indicator of the development of DME and/or PDR as well as of DR severity progression in T2D individuals with mild retinopathy.

## 1. Introduction

Diabetic retinopathy (DR) is still one of the leading causes of blindness in the world [1]. Despite the improvement in the management of diabetes, the prevalence of vision-threatening DR, such as macular edema and proliferative retinopathy, is expected to exponentially increase [2].

Early stages of DR, initially characterized by the presence of microaneurysms and/or dot hemorrhages, can predict progression to vision-threatening retinopathy. These characteristics proved to be indicators of increased risk for microvascular complications [1]. The Early Treatment Diabetic Retinopathy Study (ETDRS) severity score is the gold standard for DR staging [3]. However, it is a labor-intensive characterization method and less sensitive in the earliest stages of the disease.

Automated image analysis of microaneurysm turnover (MAT), performed on color fundus photographs with RetmarkerDR^®^, is a non-invasive technique with proven ability to identify eyes that are at risk of DR progression [2,4]. This information is of major relevance to guide follow-up decisions and determine disease prognosis but can also be important for DR clinical trial design and patient selection.

In this 5-year longitudinal study, we explored the differences in MAT rates, including formation and disappearance rates, to validate MAT as a risk marker for retinopathy progression and development of vision-threatening complications.

## 2. Materials and Methods

A 5-year prospective, longitudinal study (ClinicalTrials.gov identifier: NCT03010397), was performed to follow 212 subjects with type 2 diabetes (T2D) and mild NPDR (level 20 or 35 on the Early Treatment Diabetic Retinopathy Study (ETDRS) severity scale). One eye per subject was included and individuals were followed annually for a 5-year period, or until they developed vision-threatening complications, such as diabetic macular edema (DME) or proliferative diabetic retinopathy (PDR) [5]. The tenets of the Declaration of Helsinki were followed, and approval was obtained from the AIBILI’s Ethics Committee for Health with the number CEC/007/16. Written informed consent was signed by each participant agreeing to participate in the study.

Exclusion criteria were: (1) glycated hemoglobin A1c (HbA1c) level > 10% (85.8 mmol/mol); (2) any intravitreal injections or previous laser treatment; (3) presence of age-related macular degeneration, glaucoma, or vitreomacular disease; (4) high ametropia (spherical equivalent greater than − 6 and +2 DPT); (5) any other systemic disease that could affect the eye, with special attention for uncontrolled systemic hypertension and history of ischemic heart disease. Eyes with a baseline central retinal thickening identifying center-involving macular edema (CIME) [6,7] were also excluded.

Registry of demographic data, such as age, duration of diabetes, and physical and biometric measures (body weight and height); blood pressure evaluation; and blood analysis with the determination of HbA1c and lipid profile were performed at the baseline visit (V0) for each participant. The remaining study visits were performed at 6 months (V1, to establish DR phenotype) [5,8], 12 months (V2), 24 months (V3), 36 months (V4), 48 months (V5), and 60 months (V6), or during last visit before treatment (in eyes that developed one of the endpoints: DME or PDR).

At all study visits, individuals underwent a complete ophthalmological examination of the study eye, including best-correct visual acuity (BCVA, using the ETDRS protocol and Precision Vision charts at 4 m), slit-lamp examination, intraocular pressure (IOP) measurement, digital 7-field color fundus photography (CFP), and OCT.

The study eye was selected at baseline according to the inclusion/exclusion criteria. When both eyes fulfilled the criteria, the eye showing the more advanced ETDRS severity level was chosen as the study eye.

### 2.1. Color Fundus Photography and ETDRS Classification

Color fundus photographs (CFP) were performed according to the ETDRS protocol and under mydriasis. The seven-field photographs were obtained at 30/35° using a Topcon TRC 50DX mydriatic camera (Topcon Medical Systems, Tokyo, Japan). The DR severity level was determined by two independent graders within the context of an experienced reading center (Coimbra Ophthalmology Reading Center—CORC, Coimbra, Portugal) and was based on the 7-field protocol according to the modified Airlie House classification of diabetic retinopathy used by the Early Treatment Diabetic Retinopathy Study (ETDRS report number 12 [9]).

### 2.2. Microaneurysm Turnover Quantification by RetmarkerDR

To perform MAT quantification, additional 45/50° 2-field images were obtained under mydriasis with the same CFP equipment and subjected to automated analyses using RetmarkerDR^®^ (Retmarker SA, Coimbra, Portugal), software that performs earmarking of MAs and red-dot-like vascular lesions in the macula (all referred to as MAs); it includes a co-registration algorithm that allows comparison within the same retina location between different visits for the same eye, as previously described [10,11]. Briefly, the algorithm computes the number of MAs for each eye, per visit, and the number of MAs that appear and/or disappear from one visit to the other, allowing calculation of the MA formation rate and the MA disappearance rate, respectively, per time interval). The MAT is computed as the sum of the MA formation and disappearance rates. The calculation of MA rates was performed considering one-year intervals: V0–V2 for the first year (162 individuals) and V5–V6 for the last year of the study (127 individuals).

### 2.3. DR Severity Progression—ETDRS Step Change

Changes in ETDRS steps were determined as the difference between levels of ETDRS at baseline and at the 5-year follow-up and classified as improvement or worsening according to the reduction or increase in the retinopathy severity level. Classification included “2-step improvement”, “1-step improvement”, “maintenance”, “1-step worsening”, and “2-or-more-step worsening”. For purposes of statistical analysis, we considered worsening 1-or-more-step worsening vs. maintenance or improvement.

### 2.4. Outcome Definition—CIME, CSME, and PDR

The outcomes considered in this study were CIME, CSME, or PDR. CIME was defined as an optical coherence tomography (OCT), central subfield retinal thickness (CRT) ≥ 290 µm in women, and ≥ 305 µm in men by the Diabetic Retinopathy Clinical Research Network [5,6,7]. CSME was identified on clinical examination as defined by the Early Treatment Diabetic Retinopathy Study group as retinal thickening within 500 µm of the center of the fovea or presence of hard exudates (with thickening of the adjacent retina) within 500 µm of the center of the fovea or thickening of at least 1 disc area located less than 1 disc diameter from the center of the fovea [10]. PDR was defined by the presence of abnormal new vessels arising from the optic nerve and retina.

### 2.5. Optical Coherence Tomography

Spectral-domain OCT was performed using the Cirrus HD-OCT 5000 (Carl Zeiss Meditec, Inc., Dublin, CA, USA). The Macular Cube 512 × 128 acquisition protocol consisting of 128 B-scans and 512 A-scans each was used to assess the subjects’ CRT collected from the standard Cirrus examination reports. Eyes with CIME were identified following the reference values established by the DRCR.net for Cirrus SD-OCT [7].

### 2.6. Statistical Analysis

Variables were summarized for each ETDRS group, 10–20 and 35, using mean, SD, median, and interquartile range. For the baseline characteristics, the χ2 test for categorical variables and the Mann–Whitney U test for continuous variables were performed for comparison of both ETDRS groups.

To assess statistical differences between the first year and the last year of the study of MAT, MA formation rate, and MA disappearance rate, the Wilcoxon signed-rank test was used. Mann–Whitney U tests were performed to compare the same measurements between groups of ETDRS, severity progression, and outcome development.

Statistical analysis was performed with Stata 16.1 (StataCorp. 2019. Stata Statistical Software: Release 16. College Station, TX: StataCorp LLC.), and a *p* value ≤ 0.05 was considered statistically significant.

## 3. Results

### 3.1. Demographic Distribution of the Population

From the 212 individuals recruited with diagnosed adult-onset T2D and ETDRS 20–35 retinopathy, 172 individuals completed the 5-year follow-up or achieved an endpoint (*n* = 27). Forty dropped out of the study (9 died, 10 were lost to follow-up, and 21 withdrew from the study). Only patients that completed the 5-year follow-up study or developed outcomes were considered in the study analysis.

Forty-eight eyes (28%) were classified with ETDRS level 20 and 124 (72%) with ETDRS level 35. Of these, 162 were evaluated for MAT after 1-year of follow-up and 127 had MAT evaluation in the last year of the study. Table 1 shows the baseline characteristics of the study population and the differences between ETDRS severity levels. When analyzing the demographic characteristics, the duration of diabetes was significantly different between the two baseline ETDRS groups (*p* = 0.011). Systemically, HbA1c levels were the only feature that presented significant differences between ETDRS levels 10–20 and 35 (*p* < 0.001). MAT, MA formation rate, and MA disappearance rate at the 1st year of the study presented statistically significant differences between ETDRS levels, 10–20 and 35 (*p* < 0.001). Neither CRT nor GCL + IPL were statistically different between ETDRS levels at baseline visit.

### 3.2. Comparison of MA Changes with Development of Vision-Threatening Complications

Analyzing the development of CIME, CSME, and/or PDR during the 5-year duration of the study, MAT, calculated on the first year, showed a significant difference between the group with the appearance of any of these endpoints and the group with no appearance of endpoint (*p* = 0.018). MAT is the sum of MA formation rate and MA disappearance rate, and considering our data, the differences occurred mainly in the MA disappearance rate in individuals that developed CSME and PDR outcomes (2.2 ± 2.9) compared with those that did not develop any outcome (1.1 ± 2.0; *p* = 0.007) (Table 2).

### 3.3. Comparison of MA Changes with DR Severity Progression over 5-Years of Follow-Up

When evaluating disease severity progression along the 5-year study period, MAT in the first year showed a significant difference between ETDRS grade changes, worsening vs. improvement or maintenance, being higher in individuals that worsened their disease level (*p* = 0.035) (Table 3). The MA formation rate, indicative of disease activity, appears to be particularly relevant in eyes in these initial stages of DR (*p* < 0.001) (Table 1).

### 3.4. Microaneurysm Changes after 5-Years of Follow-Up

When analyzing MA changes across the 5-years duration of the study, similar values were seen when comparing MAT in the first and the 5-year of follow-up (*p* = 0.060). A significant increase in the MA disappearance rate was apparent in the last year of the 5-year of follow-up (*p* < 0.001) (Table 4), suggesting that after 5-years of follow-up, the MA changes occur mainly through capillary closure.

## 4. Discussion

While the presence of fewer microaneurysms is associated with less severity of the disease according to the ETDRS severity scale [2,12], the active development and closure of microaneurysms should not be considered a benign event but an indicator of significant structural damage. Microaneurysms appear and disappear in the retina of diabetic individuals over time, disappearing by closure of the capillary network and appearing in different locations of the vascular tree, possibly associated with an up-regulation of endothelial growth factors. MAT indicates a dynamic process and reflects disease activity that has been shown to be predictive of progression or worsening of retinopathy in individuals with T2D [13,14].

The present study shows that MAT and MA formation rates are related to the development of vision-threatening complications and worsening of DR severity over a 5-year period, being significantly increased in individuals that showed severity progression when compared to those who improved or maintained their disease grade (MAT 2.9 ± 3.8 vs. 2.3 ± 4.1, respectively, *p* = 0.035). An increased MAT may therefore identify a specific phenotype, associated with DR rapid progression [8]. It is noteworthy that this increase in MAT in the initial stages of DR is mainly associated with an increase in MA formation rate, which may indicate an increase in vascular endothelial growth factor levels [15].

Another interesting result of this study was the statistically significant difference found in MA dynamics, especially MA disappearance rate, and between groups of development of any study outcome, including CIME, CSME, or PDR. These findings reinforce that capillary closure and ischemia can be the primordial pathways for DR progression as proposed by Marques et al. [6]. Histologic findings of microaneurysms in DR include pericyte loss, endothelial proliferation, and apoptosis [16]. Microaneurysms are found in close association with focal areas of capillary non-perfusion but are also related to the development of cystoid spaces due to the associated alteration in the inner blood–retinal barrier [17].

Other systemic markers should be considered when evaluating MAT-associated risk for the development of vision-threatening complications. Higher levels of HbA1c were recently shown by our group to be associated with MAT risk for developing CSME and PDR [18]. In the same study, we did not find any clear association between blood pressure levels and MAT.

On the other hand, MA disappearance rate showed a significant increase in all individuals between the first and the last year of the study (1.0 ± 2.0 vs. 1.5 ± 1.9, respectively *p* < 0.001). It was especially increased in individuals with more severe DR stages (0.4 ± 0.6 in ETDRS 10–20 vs. 1.6 ± 2.4 in ETDRS level 35, *p* < 0.001. Microaneurysm disappearance rate has been shown to be associated with vessel closure [19], confirming that this feature may be an indirect measurement of capillary dropout and ischemia [20]. In fact, recently published work by our group [8] was able to demonstrate, using optical coherence tomography angiography (OCTA), that eyes that showed progression of their disease had a consistent decrease in the median values of macular vessel density (VD). Now that we can use OCTA to evaluate the retinal capillary network, this technique has also shown promising results in disease staging and identification of risk factors for progression [21], such as foveal avascular zone (FAZ) changes [22] and macular vs. periphery capillary closure [23,24]. However, OCTA is still not available in every ophthalmic clinic or primary health care unit.

The fact that MA changes in the first year are associated with the development of vision-threatening outcomes during a 5-year period highlights the value of MAT as a predictor of DR complications. This opens the possibility to use MAT as a clinically meaningful selection for clinical trials in NPDR, identifying the individuals that are at risk of progression and, therefore, improving recruitment selection and reducing the number of individuals needed to demonstrate treatment efficacy.

Limitations of this study are the inclusion of only initial stages of DR, allowing conclusions to be made only on the progression of ETDRS grades 10–35; the participation of just one clinical center, restricting the spread of the results; and the relatively well-controlled population chosen with HbA1C levels (<10%, 85.8 mmol/mol) and controlled blood pressure. Studies with a larger number of participants involving multiple centers will permit a better validation of these results in a real-world population. Nevertheless, the present work indicates that MA’s activity is associated with DR progression and vascular changes along time, in particular, capillary closure and dropout. These findings support the use of an MAT score as a useful surrogate clinical marker of risk for progression of retinopathy. More importantly, these features can be easily accessed with single-color fundus photography of the posterior pole and the use of an automatic detection software—RetmarkerDR^®^, opening its applicability not only for clinical research but also for screening purposes in any social or economic environment.

## 5. Conclusions

MAT obtained from repeated examinations using single retina fundus photographs is an indicator of DR severity progression and development of DR vision-threatening complications.

## Figures and Tables

**Table 1:** Baseline demographic and clinical characteristics of the study population per ETDRS group.

	ETDRS Groups		*p* Value (between ETDRS Groups)
	10–20	35	
**Demographics**			
**Males/Females, *n* (%)**	33 (68.8)/15 (31.3)	84 (67.7)/40 (32.3)	0.899 ^1^
**Age, mean ± SD, y**	63.2 ± 6.8, 64 (58–67.5)	62.6 ± 7.4, 63 (57.5–68)	0.619 ^2^
**Diabetes duration, mean ± SD, y**	12.0 ± 6.8, 11 (6.5–15)	15 ± 7.5, 14.5 (10–19)	**0.011 ^2^**
**Clinical Characteristics, Mean ± SD**			
**BMI, kg/m^2^**	30.1 ± 6.1, 29.1 (25.7–33.6)	30.1 ± 5.8, 29.9 (26.1–33.6)	0.874 ^2^
**HbA1c, %**	6.8 ± 1.0, 6.5 (6.2–7.5)	7.8 ± 1.3, 7.7 (6.7–8.8)	**<0.001 ^2^**
**HbA1c, % (mean, mmol/mol)**	50.8	61.7	
**Total cholesterol, mg/dL**	183.3 ± 37.9, 175.5 (155–214)	184.3 ± 39.4, 185 (159–209)	0.694 ^2^
**HDL cholesterol, mg/dL**	47.4 ± 9.2, 47 (42–53)	47.5 ± 11.8, 46.5 (40–54)	0.671 ^2^
**LDL cholesterol, mg/dL**	124.0 ± 30.9, 118 (105–146)	122.5 ± 32.1, 119 (102–140)	0.851 ^2^
**Triglycerides, mg/dL**	154.0 ± 69.6, 136.5 (104–193)	171.2 ± 101.1, 145.5 (105–196)	0.506 ^2^
**Systolic BP, mmHg**	138.4 ± 14.4, 139.5 (130–147.5)	136.3 ± 16.4, 138 (124.5–146.5)	0.432 ^2^
**Diastolic BP, mmHg**	71.5 ± 7.8, 72 (64–80)	71.6 ± 8.7, 70 (65–77)	0.808 ^2^
**Ocular Characteristics, Mean ± SD**			
**BCVA, letters**	85.8 ± 3.8, 85.5 (84–88)	85.5 ± 3.9, 85 (84–89)	0.617 ^2^
**MAT, no. per 12 months**	0.7 ± 0.9, 0 (0–1)	3.3 ± 4.5, 2 (1–4)	**<0.001 ^2^**
**MA formation rate, no. per 12 months**	0.3 ± 0.6, 0 (0–0)	1.7 ± 2.7, 1 (0–2)	**<0.001 ^2^**
**MA disappearance rate, no. per 12 months**	0.4 ± 0.6, 0 (0–1)	1.6 ± 2.4, 1 (0–2)	**<0.001 ^2^**
**CRT, µm**	276.7 ± 25.1, 277 (261.5–290)	269.6 ± 25.1, 267 (253–287)	0.069 ^2^
**GCL + IPL Average thickness, µm**	81.2 ± 7.5, 82 (76.5–86)	80.8 ± 7.2, 81 (75–85)	0.592 ^2^

Bold values represent statistically significant alterations, with *p* < 0.05. ^1^ χ2 test; ^2^ Mann–Whitney U test. BCVA: best-corrected visual acuity; BMI: body mass index; CRT: central retinal thickness; CSF: central Subfield; ETDRS: Early Treatment Diabetic Retinopathy Study; GCL: ganglion cell layer; HbA1c: glycated hemoglobin; HDL high-density lipoprotein; IPL: inner plexiform layer; LDL: low-density lipoprotein; MA: microaneurysm.

**Table 2:** Comparison of MA changes with the development of vision-threatening complications.

Outcome (DME and/or PDR)	MA Turnover 12 m (Mean ± SD Median [Q1–Q3])	MA Formation Rate 12 m (Mean ± SD Median [Q1–Q3])	MA Disappearance Rate 12 m (Mean ± SD Median [Q1–Q3])
Outcome (*n* = 25)	4.6 ± 6.6, 2 (1–5)	2.4 ± 4.0, 1 (0–3)	2.2 ± 2.9, 1 (0–2)
No Outcome (*n* = 137)	2.2 ± 3.2, 1 (0–2)	1.1 ± 1.9, 0 (0–1)	1.1 ± 2.0, 0 (0–1)
*p* value *	0.018 *	0.086	0.007 *

* and bold values represent statistically significant alterations, with *p* < 0.05, Mann–Whitney U test. Outcome = DME and/or PDR.

**Table 3:** Association of MA changes with DR severity progression over 5-years of follow-up.

ETDRS Severity Changes	MA Turnover 12 m (Mean ± SD Median [Q1–Q3])	MA Formation Rate 12 m (Mean ± SD Median [Q1–Q3])	MA Disappearance Rate 12 m (Mean ± SD Median [Q1–Q3])
Worsening (*n* = 61) (≥1 or 2 steps progression)	2.9 ± 3.8, 2 (1–4)	1.6 ± 2.2, 1 (0–3)	1.3 ± 2.1, 1 (0–2)
Improvement or Maintenance (*n* = 101)	2.3 ± 4.1, 1 (0–2)	1.1 ± 2.4, 0 (0–1)	1.2 ± 2.2, 0 (0–1)
*p* value *	0.035 *	0.033 *	0.316

* and bold values represent statistically significant alterations, with *p* < 0.05. Mann–Whitney U test. Worsening = 1-or more-step worsening in ETDRS classification.

**Table 4:** Comparison between MAT, formation, and disappearance rate in the first year and last year of the 5-year period of follow-up.

	0–12 M (n = 162) (Mean ± SD Median Q1–Q3)	48–60 M (n = 127) (Mean ± SD Median Q1–Q3)	*p* Value *
**MAT (*n* = 121)**	2.0 ± 3.2, 1 (0–2)	2.3 ± 3.1, 1 (0–3)	0.060
**MA Formation Rate (*n* = 121)**	1.0 ± 1.7, 0 (0–1)	0.8 ± 1.6, 0 (0–1)	0.089
**MA Disappearance Rate (*n* = 121)**	1. 0 ± 2.0, 0 (0–1)	1.5 ± 1.9, 1(0–2)	**<0.001**

Bold values represent statistically significant alterations, with *p* < 0.05. Wilcoxon signed-rank test.

## Data Availability

Data will be available upon request to the correspondent author.
